# Coupling Methylammonium and Formamidinium Cations with Halide Anions: Hybrid Orbitals, Hydrogen Bonding, and the Role of Dynamics

**DOI:** 10.1021/acs.jpcc.1c08932

**Published:** 2021-11-11

**Authors:** Chinnathambi Kamal, Dirk Hauschild, Linsey Seitz, Ralph Steininger, Wanli Yang, Clemens Heske, Lothar Weinhardt, Michael Odelius

**Affiliations:** † Department of Physics, Stockholm University, AlbaNova University Center, SE-106 91 Stockholm, Sweden; ‡ Theory and Simulations Laboratory, HRDS, Raja Ramanna Centre for Advanced Technology, Indore 452013, India; § Homi Bhabha National Institute, Training School Complex, Anushakti Nagar, Mumbai 400094, India; ∥ Institute for Photon Science and Synchrotron Radiation (IPS), Karlsruhe Institute of Technology (KIT), 76344 Eggenstein-Leopoldshafen, Germany; ⊥ Institute for Chemical Technology and Polymer Chemistry, Karlsruhe Institute of Technology (KIT), 76128 Karlsruhe, Germany; # Department of Chemistry and Biochemistry, University of Nevada Las Vegas (UNLV), Las Vegas, Nevada 89154-4003, United States; ∇ Department of Chemical and Biological Engineering, Northwestern University, Evanston, Illinois 60208, United States; ○ Advanced Light Source (ALS), Lawrence Berkeley National Laboratory, Berkeley, California 94720, United States

## Abstract

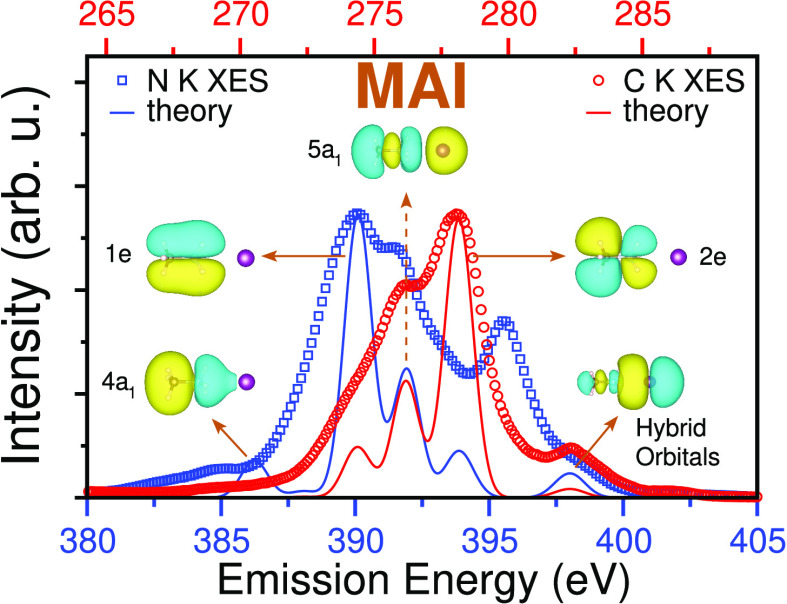

The electronic structures of four precursors for organic–inorganic hybrid perovskites, namely, methylammonium chloride and iodide, as well as formamidinium bromide and iodide, are investigated by X-ray emission (XE) spectroscopy at the carbon and nitrogen K-edges. The XE spectra are analyzed based on density functional theory calculations. We simulate the XE spectra at the Kohn–Sham level for ground-state geometries and carry out detailed analyses of the molecular orbitals and the electronic density of states to give a thorough understanding of the spectra. Major parts of the spectra can be described by the model of the corresponding isolated organic cation, whereas high-emission energy peaks in the nitrogen K-edge XE spectra arise from electronic transitions involving hybrids of the molecular and atomic orbitals of the cations and halides, respectively. We find that the interaction of the methylammonium cation is stronger with the chlorine than with the iodine anion. Furthermore, our detailed theoretical analysis highlights the strong influence of ultrafast proton dynamics in the core-excited states, which is an intrinsic effect of the XE process. The inclusion of this effect is necessary for an accurate description of the experimental nitrogen K-edge X-ray emission spectra and gives information on the hydrogen-bonding strengths in the different precursor materials.

## Introduction

Since the realization of organic–inorganic hybrid lead halide perovskite (LHP) solar cells,^1^ the achieved power conversion efficiencies have steadily increased, most recently, surpassing 25%.^2^ In spite of stability and environmental problems,^3,4^ substantial research efforts have been focused on LHPs, in particular, including methylammonium (MA) lead tri-iodide and tri-bromide (CH_3_NH_3_PbI_3_ and CH_3_NH_3_PbBr_3_), as well as their formamidinium (FA) counterparts (CH(NH_2_)_2_PbX_3_, X = I, Br). Such MA- and FA-based devices show excellent electronic, optical, and transport properties.^5−10^ Their properties can be tuned by the various choices of the cation (A) and the halide anion (X) in the general APbX_3_ LHP structure, as well as by varying the chemical composition of their binary, ternary, or quaternary mixtures.^7−10^ There are few reports on the hydrogen-bonding interaction between the organic cations and the lead halide networks and its influence on the properties of LHP.^11−14^ Despite a substantial number of experimental and theoretical investigations, key questions remain unanswered. In particular: What is the role of the organic cations and how do they interact with the complex inorganic network?

To shed light on this question, simplified systems, such as the perovskite’s precursors, MAX and FAX (with X = Cl, Br, I), promise to be useful models to develop an understanding of the interaction between the organic cations and the halide anions. The hydrogen bonding and other interactions in the precursor materials will resemble those in LHP materials, and the dependence on the choice of halide ions can be investigated. To study such model systems, X-ray spectroscopy is a natural choice, as it probes the element-specific contributions and can be used to obtain spectral fingerprints of selectively excited functional groups relating to a specific chemical structure. Especially, for nitrogen and other first-row elements, K-edge X-ray emission (XE) and absorption (XA) spectroscopies, as well as resonant inelastic soft X-ray scattering (RIXS), have been shown to provide unique information about hydrogen bonds (donation/acceptance, the influence of molecular environments, the signatures of NH_3_ and NH_4_^+^, etc.) and their dynamic influence on local electronic structures (orbital mixing, shape resonances, dipole–dipole interaction, vibronic coupling, etc.).^15−21^ As shown in the present study, the precursors, i.e., the “building blocks” of LHPs, give insights into the crucial interaction of organic cations with the halides, making it possible to improve the description of the more complex LHPs using these model systems. In fact, the XE spectra suggest that the organic cation *A* contributes to the occupied levels in the valence band and that its orbitals mix with those of the inorganic anion X.

We note that the existing literature on MAX and FAX is mainly devoted to structural, vibrational, and thermodynamic properties^22−28^ but lacks information about their electronic structures (which is crucial for an optimization of associated LHPs). Thus, in the present study, we carry out a detailed investigation of the nitrogen and carbon K-edge XE spectra of the LHP precursor materials MAX (X = Cl, I) and FAX (X = Br, I), in combination with density functional theory (DFT) electronic structure calculations. By varying both anions and cations, we can access detailed mechanistic insights, which further support XE studies of the LHP material.^29^ The influence of hydrogen bonding on the electronic structure has also been investigated with a combination of nitrogen K-edge XA spectroscopy and sampling of XA spectra from Born–Oppenheimer molecular dynamics (BOMD) simulations.^30^ Detailed analyses of molecular orbitals and electronic density of states have been carried out to identify and assign the character of the underlying orbitals that contribute to the XE spectra. We also show the formation of hybrid orbitals that couple the organic cation and the halide anion and study their contributions to the most pertinent spectral features. In addition, we report a strong influence of the hydrogen bonds on the ultrafast proton dynamics in core-excited states of the precursor materials.

## Experimental Section

Powder materials were obtained from Sigma-Aldrich (MAI, FAI, and FABr with purities of 98%) and VWR (MACl with a purity of 99%). The powders were pressed (2–3 tons/cm^2^) into pellets in air, mounted on a sample holder, and immediately transferred into the vacuum chamber of the solid and liquid spectroscopic analysis (SALSA) roll-up experimental station^31^ at the open port of beamline 8.0.1.2 of the advanced light source (ALS). XE spectra were collected using a high-transmission soft X-ray spectrometer.^32^ To minimize beam-induced changes of the spectra, we scanned the samples under the X-ray beam with 600 μm/s, which corresponded to an exposure of 50 ms for a given sample spot (X-ray spot size ∼ 30 × 150 μm^2^). Beam-induced changes were characterized in a series of measurements with different scanning speeds (see Figure S1). For the calibration of the N and C *K*-emission energy axes, we used elastically scattered (Rayleigh) lines, calibrated with a N_2_ gas phase^33^ and highly ordered pyrolytic graphite (HOPG)^34^ reference XA spectra, respectively. The absolute uncertainty of the emission energy axis is ∼0.2 eV, while the relative uncertainty between different measurements is ∼0.05 eV.

## Computational Details

To understand the origin of the spectral differences and gain microscopic insights, periodic DFT^35,36^ calculations within the generalized gradient approximation (given by Perdew–Burke–Ernzerhof (PBE))^37^ for the exchange–correlation functional, including a van der Waals correction using the Grimme’s D3 method,^38^ were performed in the CP2K package.^39^ The geometry optimizations were carried out using the Gaussian plane wave (GPW) method^40^ with Goedecker–Teter–Hutter (GTH) pseudopotentials^41^ and TZVP-MOLOPT-GTH basis sets.^42^ An energy cutoff of 600 Ry was employed. The starting supercell geometries for MACl (3 × 3 × 3), MAI (3 × 3 × 2), and FAI (3 × 1 × 2) were built using the experimental geometries of the room-temperature phases reported in the literature.^22−24^ All electronic structure calculations were performed at 0 K temperature and at the Γ point of the supercells.

Simulations of N and C K-edge XE spectra for optimized ground-state geometries (represented as geometries at time *t* = 0) were carried out using the core-level spectroscopy^43^ implementation in CP2K within the Gaussian augmented plane wave (GAPW) method,^44^ which allows for a mixed pseudopotential (GTH with TZVP-MOLOPT-GTH basis for halogens) and an all-electron (with 6-311++G2d2p basis sets for H, C, N atoms) description, thereby accessing selected core levels. The calculated discrete spectra were convoluted with a Gaussian function with a broadening parameter (σ) of 0.50 eV (corresponding to a full-width at half maximum of 1.18 eV) to compare them with the experimental spectra.

To account for dynamic effects of the XE process within the framework of classical nuclei, we carried out BOMD with the GPW method in the presence of a core hole within the *Z* + 1 approximation (since forces are not presently implemented for core-ionized/excited states with GAPW in CP2K). We used a short time step of 0.1 fs to capture the ultrafast dynamics of the protons that are mainly involved in the interaction between the halide anions and organic cations through the hydrogen bonds. The spectra were sampled at 1 fs intervals along the trajectory of up to 30 fs and then averaged with a weight factor e^–*t*/τ^, where τ is the core-hole lifetime of 5.8 fs for the N 1s core-excited state.^45^ The calculations were performed for one N atom in the MA systems and for two N atoms in the FA systems. We used VESTA software^46^ to plot geometries and molecular orbitals.

## Results and Discussion

**Figure 1:**
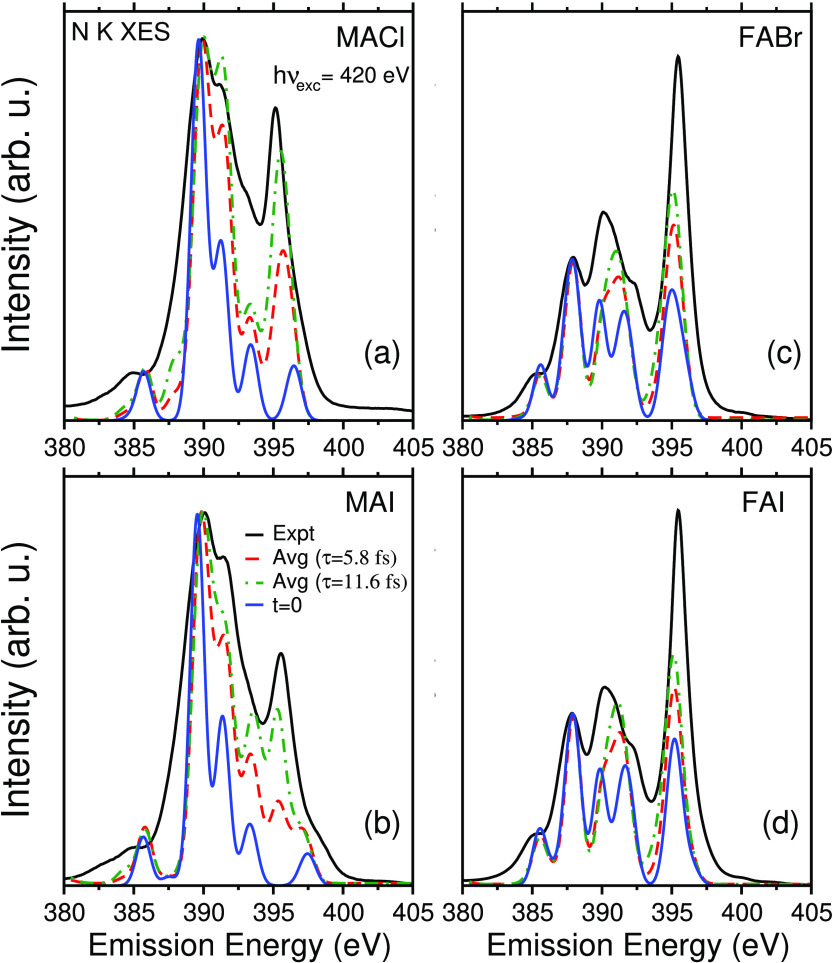
Experimental nonresonant N K-edge XE spectra for the LHP precursor materials (a) MACl, (b) MAI, (c) FABr, and (d) FAI (solid black curves, *h*ν_exc_ = 420 eV) are compared with calculated spectra for ground-state geometries (solid blue curves, denoted as *t* = 0). Core-hole lifetime (τ)-averaged spectra (τ = 5.8 and 11.6 fs with dashed red and green curves, respectively), which include core-hole dynamics, are also shown.

The experimental nonresonant (*h*ν_exc_ = 420 eV) N K-edge XE spectra for MACl, MAI, FABr, and FAI are presented in Figure 1a–d, respectively (solid black curves). The spectra for MACl and MAI are similar; both have a strong peak at ∼390 eV with a shoulder on the high-emission energy side and a small feature at 385 eV. A third peak is found at ∼395.1 eV for MaCl, which is shifted to ∼395.5 eV and reduced in relative intensity for MAI. In addition, MAI shows a shoulder at ∼398.5 eV. The N K-edge XE spectra of FABr and FAI are found to be almost identical to each other, with a sharp intense peak at ∼395 eV. They also show a peak at ∼390 eV with additional peaks on both sides. Moderate and weak shoulders at ∼385 and ∼400 eV, respectively, are also found. The corresponding experimental and theoretical C K-edge XE spectra for the studied materials are shown in Figure S2 and are briefly discussed in the Supporting Information.

**Figure 2:**
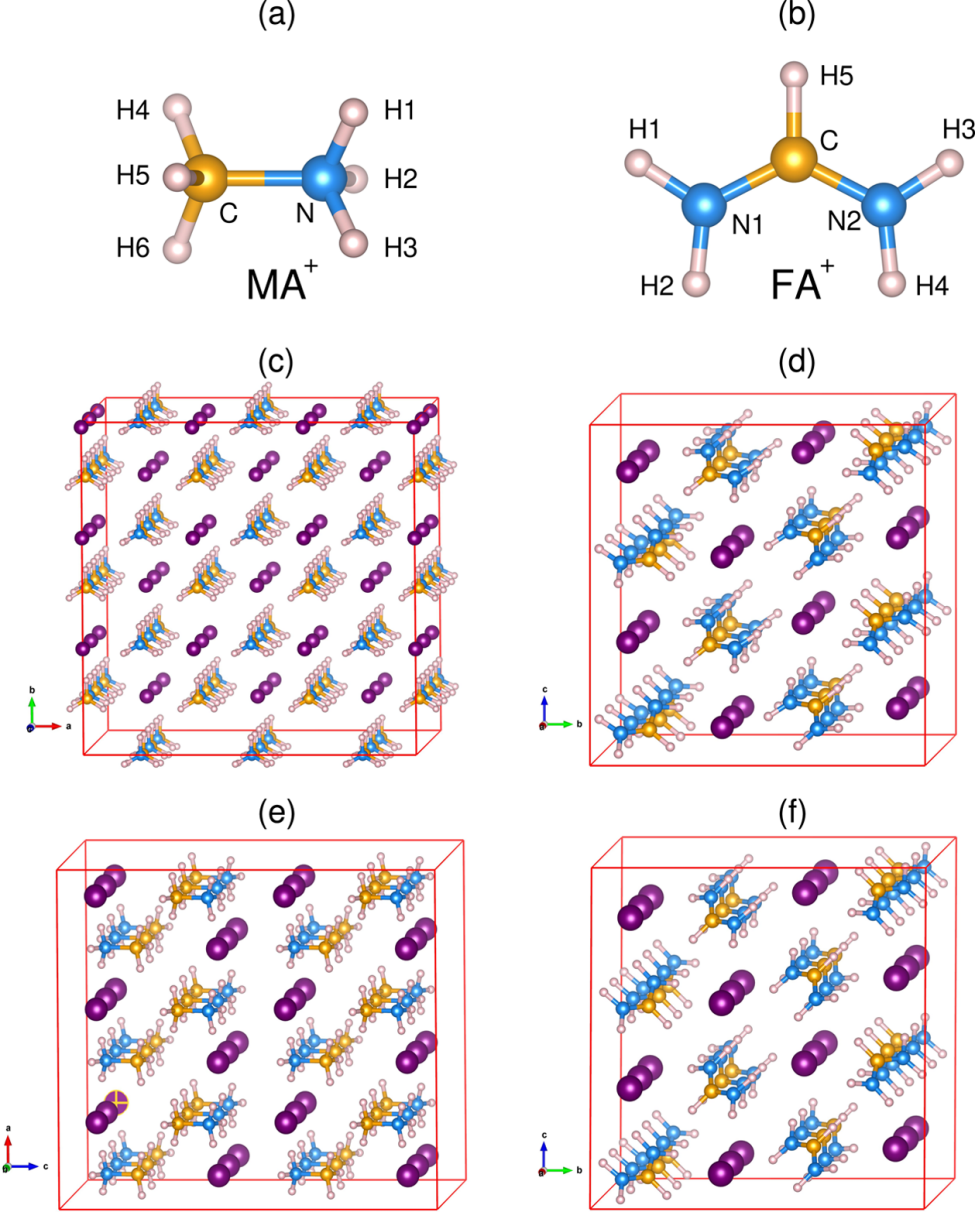
Optimized geometries of (a) MA^+^ and (b) FA^+^ molecules, as well as of solid precursors: (c) MACl, (d) FABr, (e) MAI, and (f) FAI.

First, we discuss the structural models used in our XE spectrum simulations and then compare our calculated structural parameters with experimental results available in the literature. The optimized geometries obtained for (a) MACl (tetragonal, *P*4/*nmm*), (b) MAI (tetragonal, *P*4/*nmm*), (c) FABr (monoclinic, *P*2_1_/*c*), and (d) FAI (monoclinic, *P*2_1_/*c*) in supercell models are given in Figure 2. The corresponding data for the lattice parameters and bond distances (N–H, N–C, and H···X, with X = Cl, Br, I) are summarized in Table 1. The calculated lattice parameters match quite well with the experimental data for MACl, MAI, and FAI,^22−24^ with a maximum deviation of ∼2.6 % for the lattice constant *c* in MAI. There is a clear asymmetry in N–H bond distances in both MAI and MACl, with two longer and one shorter N–H distances in MAI, whereas one longer and two shorter distances are observed in MACl. Though the N–C bond is oriented along the *c*-axis in both MACl and MAI, the relative positions of the Cl^–^/I^–^ ions are different: I^–^ ions lie along the line of the N–C bond, while the Cl^–^ ions do not. As shown in Figure S3, this has only a negligible influence on the XE spectra.

**Table 1:** Optimized Geometric Parameters for the Precursor Materials, Obtained from DFT-Based Electronic Structure Calculations with a PBE XC Functional^a^

		bond distances (Å) proton			
system	lattice parameters (Å, deg)	N–H	N–C	H···X	proton dynamics in the core-excited state
MACl	*a* = *b* = 17.912 (18.12^22^)	1.059 (H1)	1.493	2.009	H3 moves first,
	*c* = 15.202 (15.15^22^)	1.039 (H2)		2.263, 2.915	H1 and H2 follow
	α = β = γ = 90	1.039 (H3)		2.263, 2.915	
MAI	*a* = *b* = 15.350 (15.382^23^)	1.049 (H1)	1.486	2.486	H1 and H2 move,
	*c* = 17.570 (18.036^23^)	1.049 (H2)		2.487	H3 moves only slightly
	α = β = γ = 90	1.035 (H3)		2.927	
FABr	*a* = 13.888, *b* = 13.158	1.029 (H1)	1.313	2.417	H1 moves slowly,
	*c* = 13.339, α = γ = 90	1.023 (H2)		2.689	H2 stays almost in place
	β = 94.92	1.034 (H3)	1.317	2.317	H3 moves faster,
		1.025 (H4)		2.646	H4 stays almost in place
FAI	*a* = 14.324 (14.463^24^)	1.031 (H1)	1.313	2.574	H1 moves,
	*b* = 13.678 (13.776^24^)	1.023 (H2)		3.020	H2 stays almost in place
	*c* = 13.946 (14.022^24^)	1.032 (H3)	1.317	2.545	H3 moves,
	α = γ = 90, β = 97.91 (98.06^24^)	1.023 (H4)		2.959	H4 stays almost in place

a Experimental values in parentheses are from refs (22−24). The hydrogen atoms H1, H2, and H3 are bound to N in MACl and MAI. Similarly, H1 and H2 (H3 and H4) are attached to N1 (N2) of FABr and FAI. In addition, a qualitative assessment of the influence of proton dynamics in the core-excited state is added.

In FAI, the N–H bond distances associated with the two nitrogen atoms (N1 and N2) are nearly the same. For FABr, in contrast, the N–H and hydrogen bond distances^47^ associated with the N1 and N2 atoms differ more strongly. The lattice constants of FAI are also slightly larger compared to those of FABr since the ionic radius of I^–^ is larger than that of Br^–^. The hydrogen bonding clearly shows a strong dependence on the halogen (X). This is due to the decrease and increase in the halogen’s electronegativity and ionic radius (Cl^–^: 1.67, Br^–^: 1.82, and I^–^: 2.06 Å in crystals^48^), respectively, with increasing nuclear charge of the halogen ion. We will discuss later the influence of the asymmetry on the dynamics in the core-excited state.

The calculated N K-edge XE spectra for ground-state geometries of the precursor materials (solid blue curves, denoted as *t* = 0) are compared with the experimental spectra in Figure 1. The theoretical spectra are shifted rigidly by 20.5 eV for all four precursor materials to allow for a better comparison with experimental data and identify important spectral features and the molecular orbitals involved in the transitions. This procedure is used to account for an underestimation of about 5% in the emission energy obtained from DFT calculations using the ground-state Kohn–Sham orbitals. The overall shapes and energy widths of the calculated spectra for the optimized ground-state geometries match reasonably well with the experimental spectra. However, a few significant features are not well reproduced in the calculations. Most prominently, the sharp peak at ∼395.1 or ∼395.5 eV in the experimental spectra of MACl and MAI, respectively, is completely missing. In addition, there is a clear disagreement in the intensity of the high-emission energy shoulder of the ∼390 eV peak. In agreement with the experiment, the theoretical MACl spectra show a peak at ∼397 eV, slightly larger in intensity and shifted to lower emission energies compared to the corresponding peak in MAI.

The theoretical N K XE spectra for FABr and FAI look almost identical to each other but differ from the experimental results. Again, the intensities of the peaks at ∼395 and ∼390 eV are grossly underestimated in the theoretical spectra. As shown below, dynamic effects in the core-excited state need to be included to account for the above-mentioned deficiencies.

Note that we have performed geometry optimization for MACl and MAI in two tetragonal phases that differ in the relative position of the halide anion with respect to the N–C bond of the MA^+^ cations. The calculations indicate that these two alternate structures are energetically less favorable by about 130 meV per formula unit than the geometries given in Figure 2. This result corroborates the experimental data.^22,23^ Importantly, the relative orientation of the organic cations and the halide anions is also expected to be rigid even at room temperature since the energy difference is much higher than the thermal energy (∼25 meV). To probe the influence of this aspect on the spectra, we have also simulated the XE spectra for MAI and MACl in the respective phase optimized for the other compound. We find that the thus-simulated XE spectra of MACl and MAI are quite similar (Figure S3), suggesting that the interaction between MA^+^ and the halide ions is nearly the same for these two phases. In addition, this result also indicates that the effect of dynamical averaging of organic molecular orientation on the XE spectra at a finite temperature may not be essential to include in the description.

**Figure 3:**
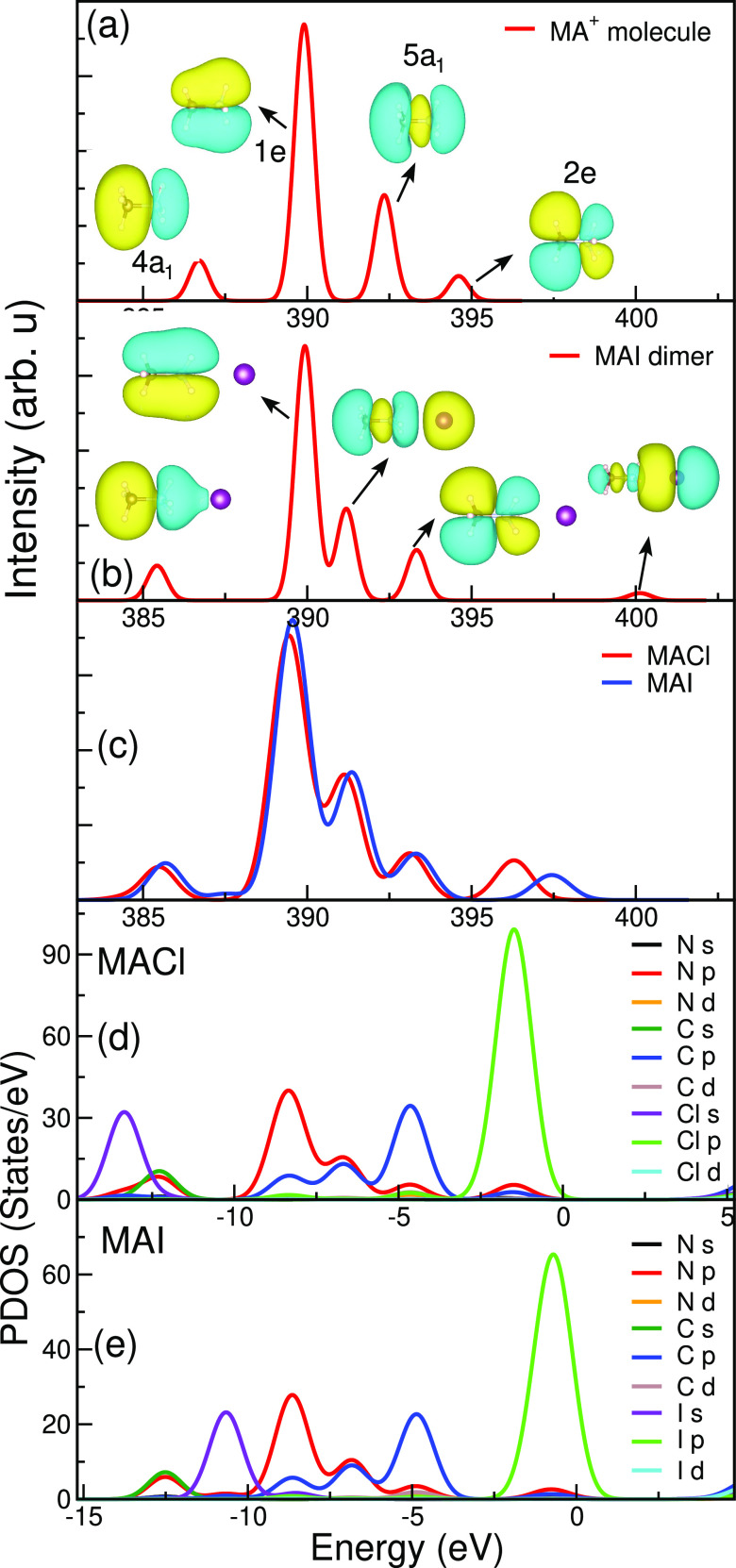
Calculated nitrogen K-edge XE spectra for (a) MA^+^ molecule, (b) MAI dimer, (c) MACl and MAI solids. The projected density of states (PDOS) for (d) MACl and (e) MAI solids.

**Figure 4:**
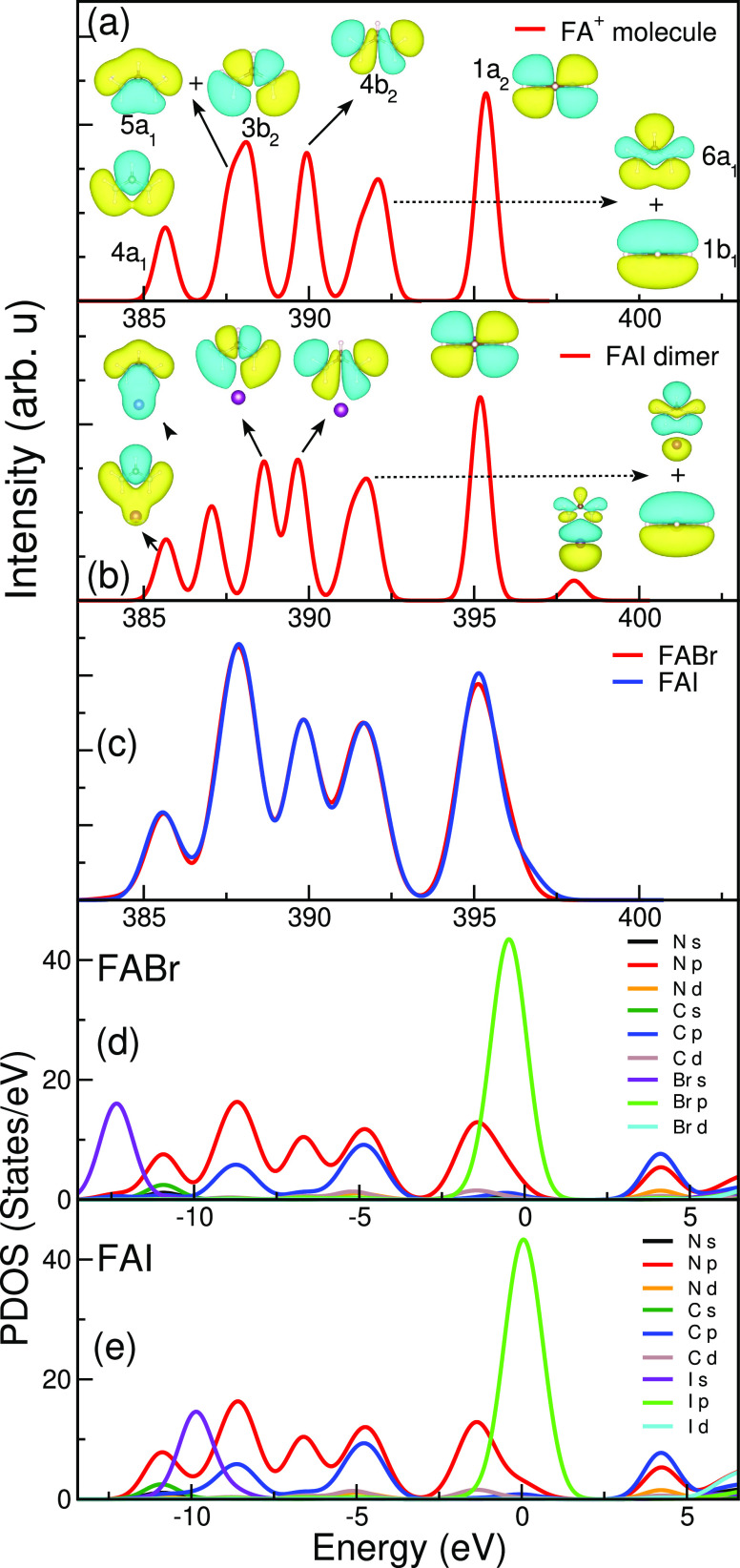
Calculated nitrogen K-edge XE spectra for (a) FA^+^ molecule, (b) FAI dimer, (c) FABr and FAI solids. The projected density of states (PDOS) for (d) FABr and (e) FAI solids.

To assign and explain the similarities and differences in the XE spectra of the MA- and FA-based precursor materials, we analyze the character of the molecular orbitals involved in the electronic transitions and the corresponding projected density of states (PDOS) of the KS orbitals. This will also give insights into the influence of hydrogen bonding and the spectral response to core-excited state dynamics. To gain a deeper understanding of the nature of the occupied states, we have also simulated XE spectra for the isolated MA^+^ and FA^+^ molecular ions, as well as the isolated MAI and FAI dimers, which are given in panels (a) and (b) of Figures 3 and 4, respectively. The results for these molecular systems are then compared to the MA- and FA-based solids in Figures 3c and 4c, respectively.

The N K-edge XE spectra of both MA- and FA-based systems show strong similarities with their corresponding molecular (MA^+^ and FA^+^) spectra. To identify and make a one-to-one mapping between the peaks of the solids and the molecules, the molecular character of the KS orbitals underlying the simulated spectra is crucial; we have therefore depicted the molecular orbitals in Figures 3 and 4.

For the MA-based systems (MACl and MAI), the four main peaks are due to electronic transitions from the occupied valence molecular orbitals, denoted as 4a_1_, 1e, 5a_1_, and 2e, to the N 1s core hole. The orbital symmetries (i.e., a_1_ and e) are derived from the irreducible representations of the C_3v_ point group of the isolated MA^+^ molecule. The main peak at ∼390 eV in the experimental spectra is thus due to the 1e orbital of π symmetry around the N–C bond. In comparison to the MA^+^ molecule with the MAI dimer, we notice that the 4a_1_, 5a_1_, and 2e derived orbitals experience a downward shift upon dimer formation. Furthermore, we also note the presence of an additional weak hybrid peak on the higher emission energy side in the spectrum of the MAI dimer (at ∼400 eV) and MAI solid (at ∼397.5 eV).

To compare with the spectra of the MA^+^ ion, MAI dimer, and MAI solid, our results of the PDOS of the precursor solids are given in Figure 3d,e to show the contributions of the C and N atomic orbitals to the occupied valence states. To facilitate the comparison, the energies of the PDOS in panels (d) and (e) and the XE spectra in panels (a–c) have been aligned using the N 1s core level as a reference. The energy values for the PDOS shown in panels (d) and (e) of Figures 3 and 4 are given with respect to the Fermi level. This depiction clearly indicates that the XE spectra generally follow the PDOS of the N p orbital in both the MACl and MAI materials.

The results of the PDOS and the dimer calculations suggest that hybrid orbitals are formed due to hybridization between the MA constituents (mainly from C and N p derived orbitals) and the halogen (Cl or I) atomic orbitals. In particular, the high-emission energy peak observed in the experimental spectra (∼395.1 eV for MACl and ∼395.5 eV for MAI) can now be identified (at ∼396 and ∼397.5 eV, respectively, in panel (c)). However, the intensities of these peaks in the simulated XE spectra are weaker, and their energies are slightly higher in comparison with the experimental spectra. Nevertheless, the simulated peaks obtained for the ground-state geometries (which do not include dynamical effects) thus already explain the presence of high-emission energy shoulders in the experimental spectra.

For MAI, the percentage contributions of the MA constituent atoms and I 5p orbitals to the hybrid orbital at ∼397.5 eV are 12.1 and 87.9%, respectively. For the hybrid peak in the dimer (∼400 eV in panel (b)), the contributions are 6.4 and 93.6%. These values are calculated by summing up the contributions from the atomic orbitals of all atoms to the hybrid molecular orbital. The higher the contribution from the orbitals of the organic cations to the hybrid orbitals, the stronger the orbital mixing and hence the coupling between the organic cations and the inorganic anions. The electrostatic attraction between the MA^+^ and halide ions competes with the repulsive orbital overlap; the electronic structure is relaxed by orbital mixing, which is a signature of hydrogen bonding also observed for methylammonium lead halide perovskites and aqueous ammonia.^16,29^ Thus, the hybridization responsible for the appearance of the high-emission energy peak (∼395.1 eV for MACl and ∼395.5 eV for MAI) arises primarily from the hydrogen bonding in the solid materials. Similar hybridization is observed in the MAI dimer as well. However, the contribution of the MA-derived orbital in the MAI dimer is smaller than in the solid. To be visible in the experimental XE spectra, this peak must also correspond to a (partial) filling of the lowest unoccupied molecular orbital (LUMO) of the MA^+^ ion. In contrast to the newly formed hybrid orbitals, the character of the MA-based orbitals (4a_1_, 1e, 5a_1_, and 2e) is retained upon formation of the solids. This is understandable since they only contain very small contributions from the atomic orbitals of Cl and I.

In comparison to the MAI solid, the contribution of the MA-derived orbitals (MA: 16.5%, Cl 3p: 83.5%) in the hybrid orbital of MACl is slightly larger. Thus, the coupling of the MA^+^ cation and the halide in MACl is slightly stronger compared to MAI. This result is also consistent with the geometrical analysis that shows the hydrogen bond distance in the MACl solid (shortest H···Cl = 2.009 Å) to be shorter than in the MAI solid (shortest H···I = 2.486 Å, see Table 1). In essence, the hybrid orbital can be used to describe the impact of the subtle differences in the bonding environment in the two compounds.

We have carried out a similar analysis for the FA-based systems, as presented in Figure 4. In Figure 4a, we observe five distinct peaks in the N K XE spectra of the isolated FA^+^ molecule. They are due to the electronic transitions from seven occupied valence molecular orbitals with strong N 2p orbital character, namely, 4a_1_, (5a_1_ + 3b_2_), 4b_2_, (6a_1_ + 1b_1_), and 1a_2_. Similar to the MAI dimer, an additional hybrid peak appears in the simulated XE spectra of the FAI dimer in Figure 4b on the high-emission energy side at ∼398 eV. The corresponding hybrid orbitals have a major contribution from I 5p orbitals (90.8%), in addition to the orbitals of FA (9.2%). In the case of the FABr and FAI solids, the hybrid peak in the XE spectra in Figure 4c partly overlaps with the peak of the 1a_2_ orbital to produce a weak high-emission energy shoulder. The PDOS of the precursor materials in Figure 4d,e show the major contributions of the N p orbitals to the XE spectra. We have calculated the contributions of halide and FA orbitals to the hybrid orbitals of the solids: FA-derived orbitals (18.2%) and Br 4p (81.8%) in FABr, and FA-derived orbitals (12.7%) and I 5p (87.3%) in FAI. Similar to the MA-based materials, the coupling between the organic (FA) cation and the halide anion depends on the choice of halogen: the coupling in FABr is stronger than in FAI, for which it is nevertheless significantly higher than in the FAI dimer.

This analysis guided by variations of the ion composition clearly suggests that hybridization plays an important role in understanding the experimental XE spectra and that the simple ion pairs (dimers) can already give valuable insights into the effect of the local bonding environment on the spectra (as seen in the high-emission energy peak, arising from electronic transitions involving the hybrid orbital). However, the mismatch between the theoretical and experimental spectra in Figure 1 at ∼395 eV suggests that additional effects need to be taken into account to understand the XE spectra. In the following, we will thus discuss the impact of proton dynamics on the XE spectra of MACl, MAI, FABr, and FAI.

The evolution of various bond distances and the corresponding XE spectra as a function of “snapshot time after core excitation” are summarized in Figures S4–S7 for MACl, MAI, FABr, and FAI, respectively. The core-hole lifetime-averaged spectra are then calculated from these snapshot spectra with a weight factor of e^–*t*/τ^ for the N 1s core-hole lifetime τ of 5.8 fs and are compared with the experimental spectra in Figure 1. As noticed in previous studies of the O K-edge and N K-edge XE spectra and RIXS data,^16,18,29,49,50^ the classical modeling tends to underestimate the effects of proton dynamics. Hence, here, we also included a longer lifetime average (by replacing τ with 2τ, i.e., 11.6 fs) in Figure 1.

In doing so, we observe significant improvements in the calculated spectra. The lifetime-averaged spectra of MACl and MAI show an intense peak at ∼395 eV, which is completely missing in the spectra based on the ground-state geometries. The time evolution of the spectra clearly indicates that the origin of this sharp peak is a shift of the high-emission energy peak (arising from the hybrid orbital) to lower emission energies due to ultrafast proton dynamics. Until *t* = 5.8 fs, the characters of the underlying orbitals of all four materials are retained (see Figures S4–S7), in spite of energy shifts and relative intensity changes of the corresponding peaks. These shorter time scale spectra also contribute more strongly to the lifetime-averaged spectra. The hydrogen atoms (bound to N) with shorter H···X distance in the ground-state geometries move faster compared to all other atoms, forming bonds to the halide ions at ∼11 and ∼16 fs for the MACl and MAI systems, respectively. At these geometries, the peaks in the XE spectra look quite different from those of the ground-state geometries, since the NH_3_ group in MA^+^ becomes NH_2_-like in MACl and NH-like in MAI.

To quantify the impact of proton dynamics on the hybrid orbitals and hence on the high-emission energy peak, we have calculated the changes in the contribution of the N p orbital to the hybrid orbitals. For the geometries near *t* = 5.8 fs, we find that the N p contribution increases by 4.2 and 3.4% (with respect to the corresponding ground-state geometry) in MACl and MAI, respectively. This result highlights the important role of hydrogen bonding (through organic-halide ion pairing) on the proton dynamics. It also explains why the peak at 395 eV in MACl is sharper than that in MAI.

Another major improvement achieved by including proton dynamics in the calculations is a substantial increase in the intensities of the peaks at ∼391 and ∼393 eV in both MACl and MAI, leading to a better agreement between theoretical and experimental spectra. For the FABr and FAI solid systems, we have carried out two separate core-hole dynamics simulations to account for the two N atoms present in the FA^+^ molecule. In FAI, the time evolution in the presence of a N 1s core hole is nearly the same for these two N atoms, except that the proton dynamics at the N1 site lag behind those at the N2 site (by about 2 fs, see Figure S7). This small difference is attributed to the fact that the hydrogen-bonding environments around these two N atoms are quite similar in the ground-state geometry of FAI (see Table 1). The lifetime-averaged spectra of FAI show significant improvements in the intensities of the peaks at ∼392 and ∼395 eV. Again, the improvement at ∼395 eV is due to the increase of the N p orbital contribution to the hybrid orbitals in the partially dissociated geometry. The increase is calculated to be 1.9% in FAI for the geometry near *t* = 5.8 fs.

In the case of FABr, the dynamics of the systems with a core hole at either the N1 or the N2 atoms are quite different: the proton attached to N2 moves much faster. Again, the origin of this difference can be attributed to the asymmetric hydrogen-bonding environment around these two N atoms in the ground-state geometry. This leads to different time evolutions of the spectra associated with the N1 and N2 atoms. The lifetime-averaged spectra of N2 show a significantly improved agreement of the intensities of the peaks at ∼392 and ∼395 eV. In comparison to FAI, we find a larger increase (about 5.3%) in the N p orbital contribution to the hybrid orbitals for the geometry near *t* = 5.8 fs. Note that the differences in the strengths and asymmetry of the hydrogen bonds in FABr and FAI are attributed to electronegativity/ionic radii differences between the halide anions. In contrast to the proton dynamics observed in the MA-based systems, the migration of the hydrogen atom from the FA molecule to the halide ion occurs after longer time scales, i.e., ∼19 and ∼20 fs in FABr and FAI, respectively. This can possibly be attributed to the differences in hydrogen dissociation between the NH_2_ and NH_3_ groups. We note that the good agreement between the calculations and experiments in Figure 1 is obtained with a very approximate treatment of vibrational broadening, in combination with classical BOMD simulations of the large-amplitude N–H elongation in the core-ionized state. A more detailed description of the vibrational envelopes is required to capture the broadness of the peak at ∼385 eV (associated with the bonding nature of the 4a_1_ molecular orbital). Furthermore, the sharpness of the peak at ∼395 eV is associated with a nonbonding lone-pair orbital in MA arising from proton dynamics, which requires a more elaborate quantum mechanical treatment, as previously demonstrated in small molecular systems.^51,52^

Overall, our calculations show that ultrafast proton dynamics strongly influences the XE spectra of the investigated precursor materials, in particular, the high-emission energy peak corresponding to hybrid orbitals, which couple the organic cation with the halide anion. Thus, the inclusion of the hybrid orbitals, even at the classical level, yields significant improvements in the theoretical description. This supports the analysis in related studies of the methylammonium lead halide perovskite, for which hybrid orbitals and the influence of bond dissociation have also been identified.^29^ Though our static models of the XE spectra lack the dynamical averaging of the orientation and hydrogen bonding of the MA^+^ and FA^+^ ions at a finite temperature, the dynamical evolution in the core-ionized state is shown to be essential for an accurate description of the XE process.

## Conclusions

In summary, a combined study of nitrogen and carbon K-edge X-ray emission spectroscopy and DFT-based electronic structure calculations for four different precursor materials of organic–inorganic hybrid lead halide perovskites, namely, MACl, MAI, FABr, and FAI, has been carried out. The N K-edge spectra of MACl and MAI are quite similar to each other, except near the high-emission energy peaks, which arise due to the hybridization of orbitals of the organic cation and the halide anion. The hybridization in MACl is found to be slightly stronger than that in MAI. While the FA-based XE spectra differ significantly from those of the MA-based systems, similar high-emission energy peaks are found that also correspond to FA and halide-derived hybrid orbitals. A detailed analysis of the molecular orbitals and density of states was carried out to identify and assign the character of the underlying molecular orbitals. Major parts of the XE spectra of the precursors can be correlated with their molecular counterparts, whereas the hybrid orbitals, contributing to the high-emission energy signals, cannot. Our simulations reveal the importance of hydrogen bonding in coupling organic and halide ion pairs and its role in ultrafast proton dynamics within the femtosecond relaxation time of the excited core hole. Overall, XE spectroscopy of the precursor materials provides valuable information about the interaction between the organic cations and halide anions dependent on the chosen halide. This is the first important step for understanding the interactions in organic–inorganic hybrid lead halide perovskite materials, in which the contribution to the hybrid orbitals will be even more complex due to the presence of the lead halide network. These interactions have been shown to influence the inorganic lattice and overall crystal structure.^53^

## Supporting Information Available

Beam-induced changes; nonresonant and simulated C K-edge XE spectra; and influence of geometry and proton dynamics on the core-excited state, as seen in the N K-edge XE spectra (PDF)

## Notes

Data sets generated during the current study are available from the corresponding authors on reasonable request.
