# Supplementary material for: Coupling Methylammonium and Formamidinium Cations with Halide Anions: Hybrid Orbitals, Hydrogen Bonding, and the Role of Dynamics
Source: J Phys Chem C Nanomater Interfaces. 2021 Nov 11;125(46):25917–26. doi: 10.1021/acs.jpcc.1c08932 (PMC8634158; doi:10.1021/acs.jpcc.1c08932)
Supplement: Supplementary file 1 — jp1c08932_si_001.pdf [file jp1c08932_si_001.pdf]

Supplementary Information for Coupling Methylammonium and Formamidinium Cations with Halide Anions: Hybrid Orbitals, Hydrogen Bonding, and the Role of Dynamics

Chinnathambi Kamal,[*,†,‡,¶] Dirk Hauschild,[§,‖,⊥] Linsey Seitz,[§,#] Ralph Steininger,[§] Wanli Yang,[@] Clemens Heske,[§,‖,⊥] Lothar Weinhardt,[*,§,‖,⊥] and Michael Odelius[*,†]

†*Department of Physics, Stockholm University, AlbaNova University Center, SE-106 91 Stockholm, Sweden*

‡*Theory and Simulations Laboratory, HRDS, Raja Ramanna Centre for Advanced Technology, Indore - 452013, India*

¶*Homi Bhabha National Institute, Training School Complex, Anushakti Nagar, Mumbai - 400094, India*

§*Institute for Photon Science and Synchrotron Radiation (IPS), Karlsruhe Institute of Technology (KIT), 76344 Eggenstein - Leopoldshafen, Germany*

‖*Institute for Chemical Technology and Polymer Chemistry, Karlsruhe Institute of Technology (KIT), 76128 Karlsruhe, Germany*

⊥*Department of Chemistry and Biochemistry, University of Nevada, Las Vegas (UNLV), NV 89154-4003, USA*

#*Department of Chemical and Biological Engineering, Northwestern University, Evanston, IL 60208, USA*

@*Advanced Light Source (ALS), Lawrence Berkeley National Laboratory, Berkeley, CA 94720, USA*

E-mail: ckamal@rrcat.gov.in; lothar.weinhardt@kit.edu; odelius@fysik.su.se

Beam-induced changes

To minimize beam-induced contributions to the spectra, the samples were continuously scanned under the x-ray beam. To determine an optimal scanning speed, spectra were collected with different scanning speeds. This is exemplarily shown for formamidinium bromide (FABr) in Figure S1.

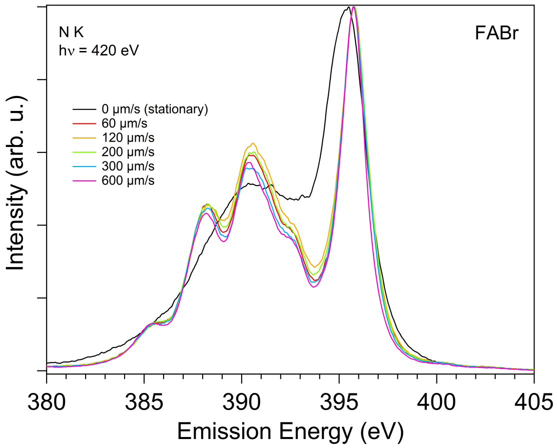

Figure S1: Non-resonant N K XE spectra of FABr for different scanning speeds under x-ray exposure. The 0 μm/s (stationary) measurement was collected for 60 sec.

The non-scanned (i.e., stationary) XE measurement exhibits a prominent peak at 395 eV and only a single broad feature at ~390 eV, which differs significantly from the scanned measurements. The spectral signature changes significantly when the exposure time for a given spot is reduced by scanning the sample under the x-ray beam. For 60 μm/s, the XE spectrum already resembles the general structure of the XE spectrum of the undamaged FABr. For scan speeds $\geq$ 300 μm/s, the spectra are essentially identical. To ensure a minimum of beam-induced changes, all measurements were performed with 600 μm/s scanning speed.

Non-resonant C K-edge XE spectra

Figure S2 gives the experimental (black) and calculated (blue) non-resonant carbon K-edge XE spectra for the precursor materials MACl (a), MAI (b), FABr (c), and FAI (d). The

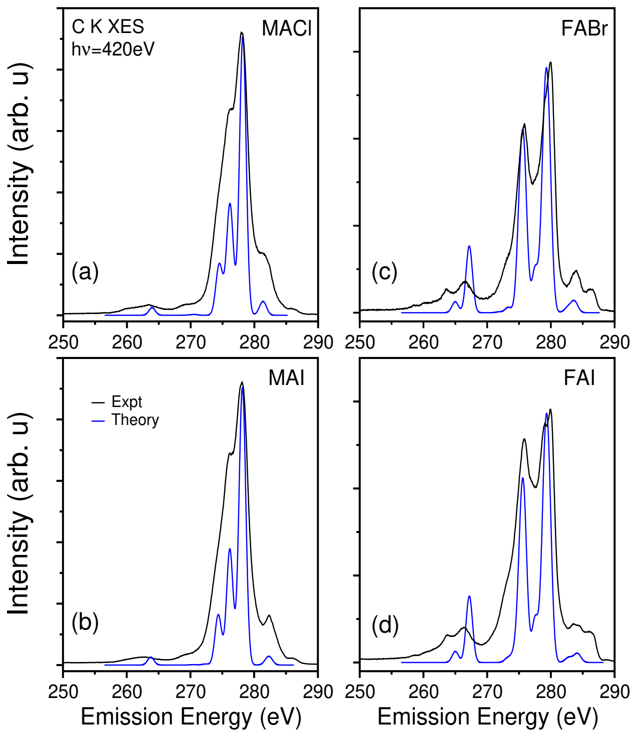

Figure S2: Experimental C K-edge X-ray emission spectra for the precursor materials: (a) MACl, (b) MAI, (c) FABr, and (d) FAI. Experimental spectra are compared with calculated spectra of optimized geometries (solid blue curves).

C K-edge XE spectra for MACl and MAI look very similar, as is the case for FABr and FAI. Both the MA and FA related spectra exhibit several features. The MA spectra exhibit a weak and broad peak at ∼264 eV and an intense and sharp feature at ∼278 eV, with a shoulder at ∼276 eV. At higher emission energies, an additional peak is observed, which is more distinct for MAI than for MACl. The FA spectra show a weak double-peak structure at low emission energies, a strong double-peak feature at ∼275 and ∼279 eV, and two broader less-intense features at ∼284 and ∼286 eV.

A comparison of the theoretical and experimental XE spectra shows a close agreement (after shifting the theoretical spectra by 18.0 eV to align them with the experimental spectra). Additionally, dynamical simulations with a core hole at the carbon atoms in MAI and FAI show fluctuations of only about ± 0.5 Å in the C–H bond distances and no dissociation up to 40 fs. Consequently, dynamic effects are less important for the C K-edge XE spectra than for the corresponding N K-edge XE spectra. The intensity of the C K-edge XE spectra follows

the C p orbital's projected density of states (PDOS) of the Kohn-Sham orbitals, which are given in panels (d) and (e) of Figures 3 and 4 in the main text. We are also able to identify the molecular orbitals contributing to the various peaks in the C K XE spectra of both MA and FA based materials. For instance, the first four low emission-energy peaks (below 280 eV) of the MA based materials are due to electronic transitions from the occupied valence molecular orbitals, with characters $4a_1$, 1e, $5a_1$ and 2e, to the C 1s core orbital; the most intense peak is due to the 2e orbital. A similar analysis can be performed for the FA based systems, which suggests that the most intense peak at ~280 eV in the C K-edge XE spectra of FA based materials is due to electronic transitions from ($6a_1$ + $1b_1$) orbitals to the C 1s core hole.

Geometry influence on the XE spectra

MA based precursors exist in two alternative tetragonal structures. Consequently, we have carried out the structural optimization of MAI in MACl's structure and vice versa to probe the influence of geometry on the spectra. The results of our calculations show that the two "opposite" geometries are energetically less favorable by about 130 meV per formula unit than the geometries given in Figure 2 of the main text, consistent with the experimentally measured geometries. The XE spectra for these geometries have also been calculated, as given in Figure S3, and indicate only a negligible influence of the structure on the spectra (compare the solid and dashed lines for each color).

Dynamical effects in the XE spectra

The evolution of the N K-edge XE spectra as a function of time, and the corresponding changes in the various bond distances, are presented in Figures S4, S5, S6 and S7 for MACl, MAI, FABr, and FAI, respectively. We have performed ab initio molecular dynamics simulations with a core hole at the N 1s level for the duration of 30 fs and simulated XE spectra for each fs. These have been used to calculate the lifetime averaged spectra given in Figure 1

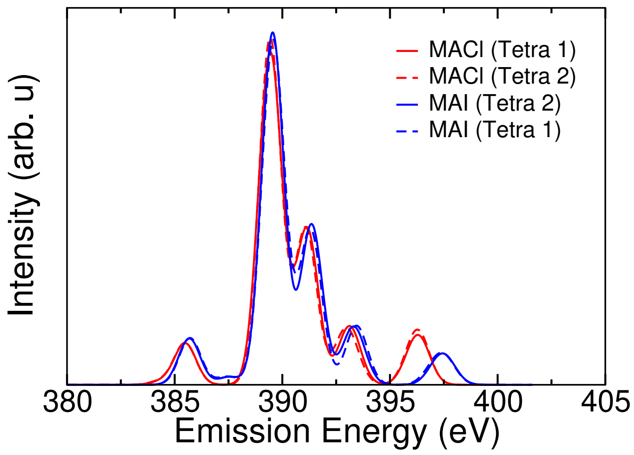

Figure S3: Theoretical N K-edge XE spectra for MACl and MAI in two different tetragonal crystal structures - the optimized structure for MACl and MAI (solid lines), as well as the structure optimized for the other compound (dashed lines).

of the manuscript. For the purpose of presentation, the individual time-stamped spectra are given only up to 20 fs in Figures S4 to S7.

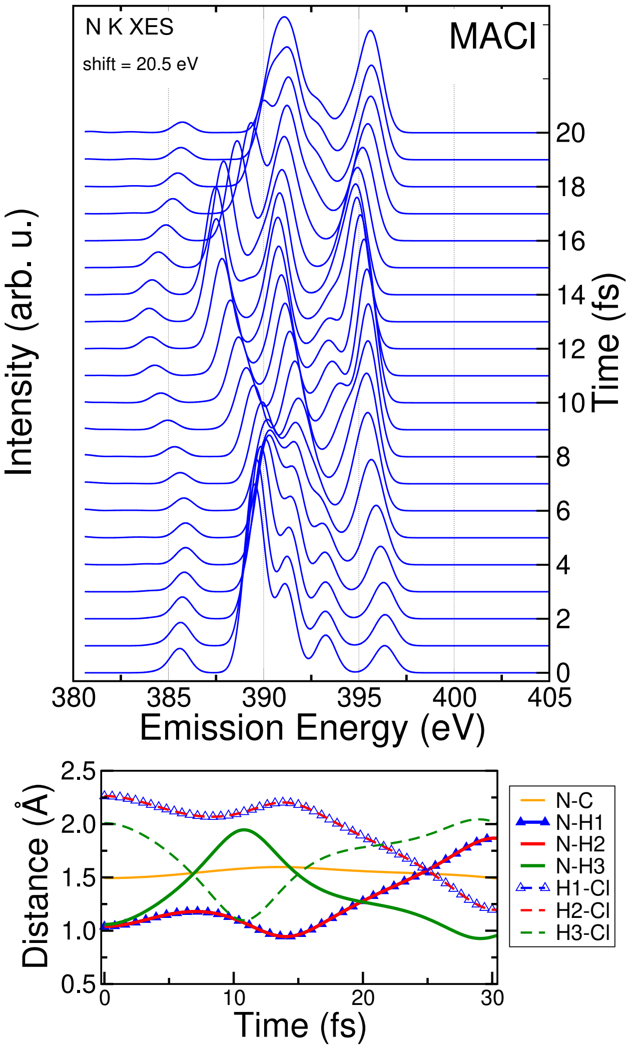

Figure S4: Evolution of X-ray emission spectra after core-hole excitation of the N atom of MACl (top), and the corresponding variations in bond length (bottom).

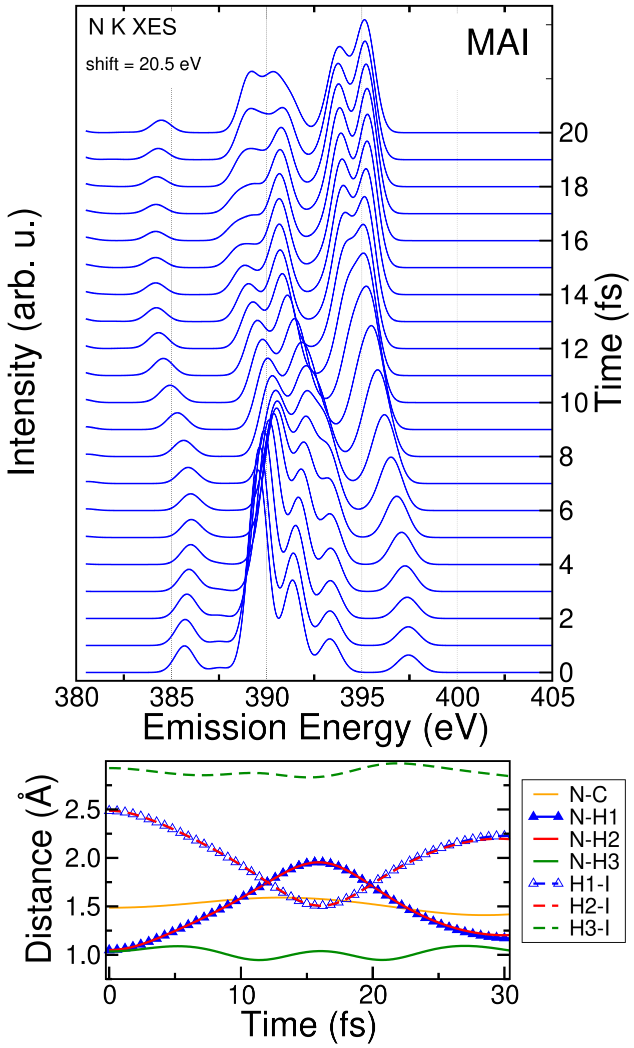

Figure S5: Evolution of X-ray emission spectra after core-hole excitation of the N atom of MAI (top), and the corresponding variations in bond length (bottom).

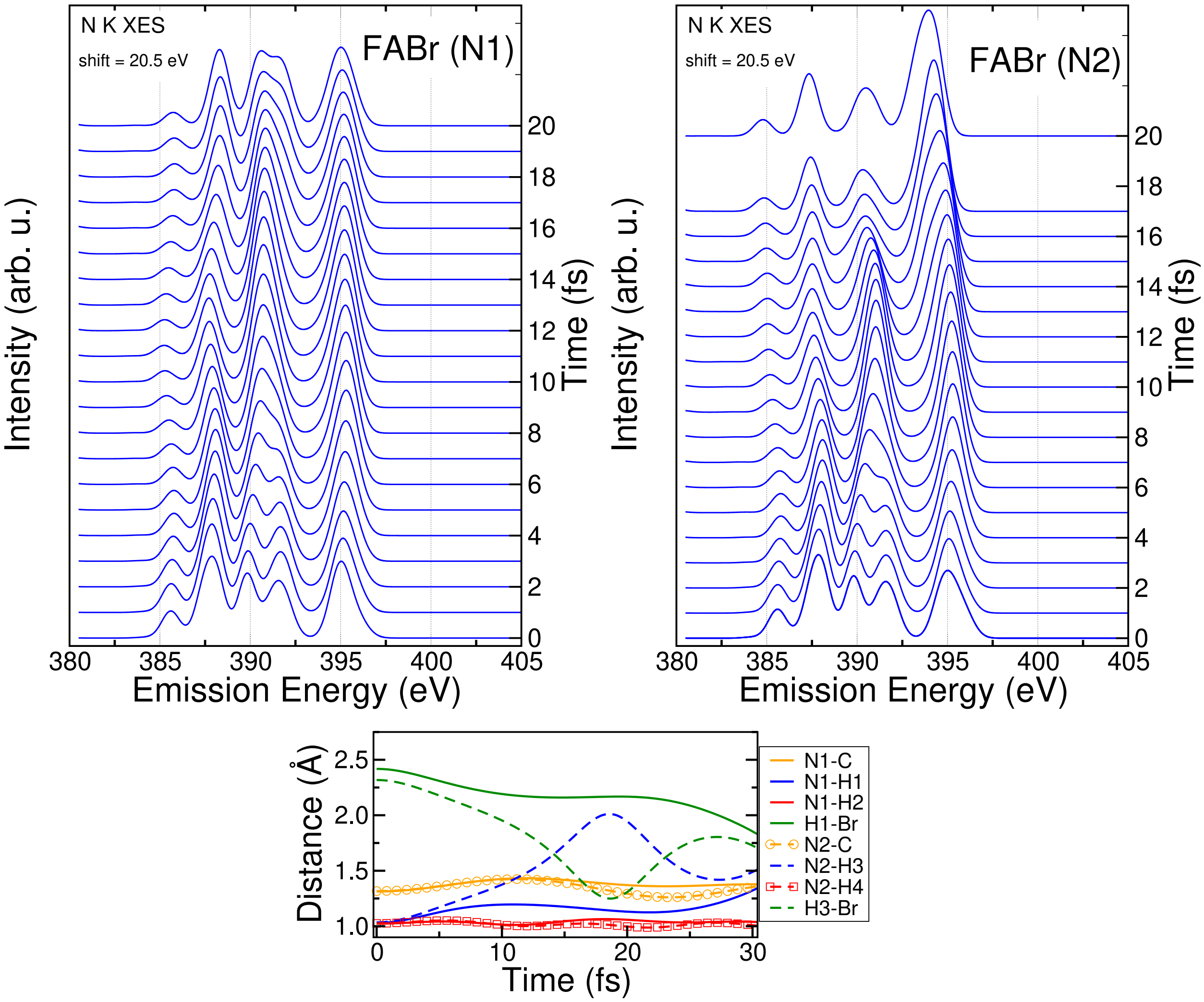

Figure S6: Evolution of X-ray emission spectra after core-hole excitation of the two different N atoms of FABr and the corresponding variations in bond length.

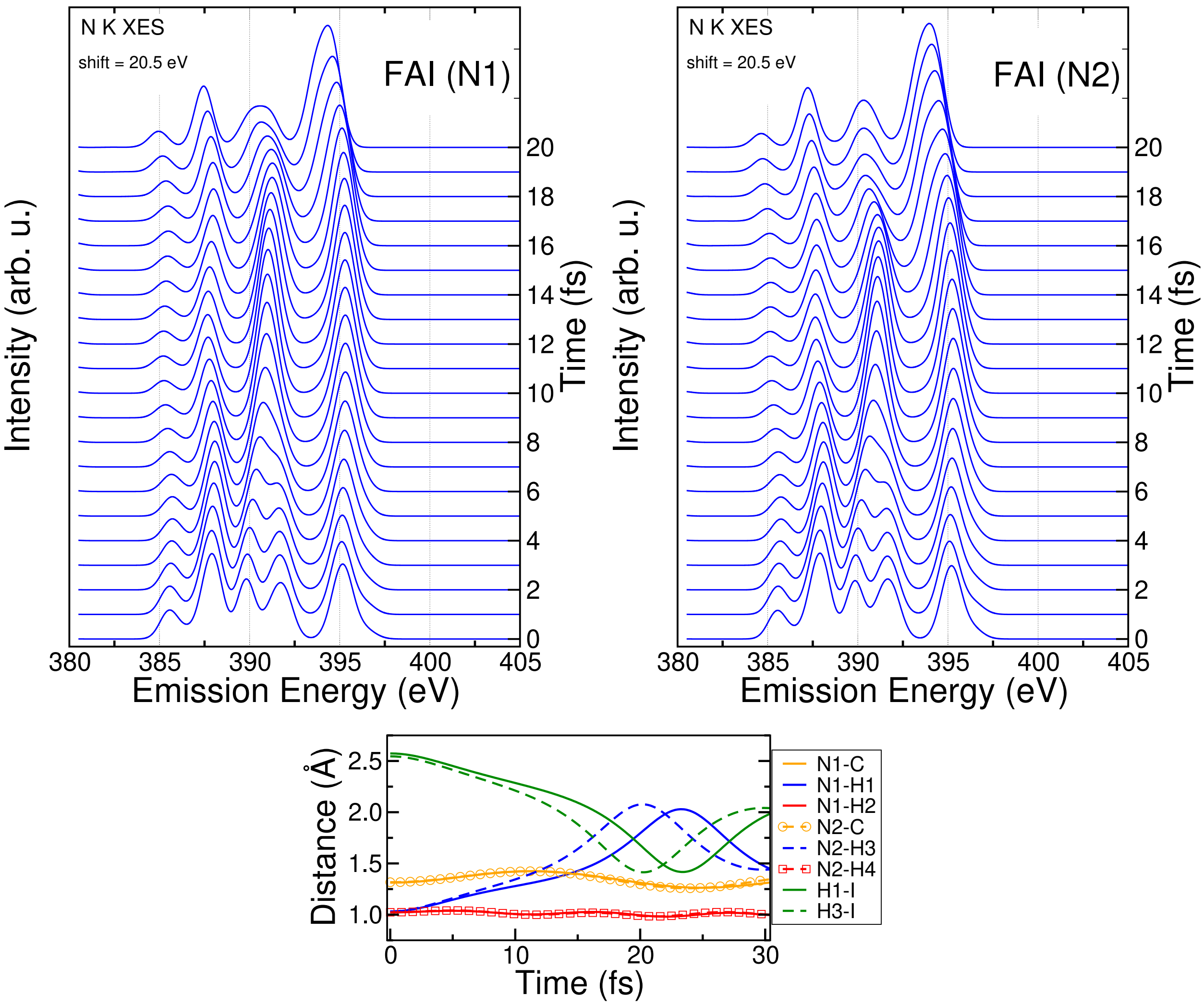

Figure S7: Evolution of X-ray emission spectra after core-hole excitation of the two different N atoms of FAI (top), and the corresponding variations in bond length (bottom).
